# Incidence of syphilis in Greenland 2010–2014: The beginning of a new epidemic?

**DOI:** 10.3402/ijch.v74.28378

**Published:** 2015-07-17

**Authors:** Nadja Albertsen, Gert Mulvad, Michael L. Pedersen

**Affiliations:** 1 Queen Ingrid Health Care Centre, Nuuk, Greenland; 2 Greenland Centre of Health Research, Institute of Nursing and Health Science, University of Greenland, Nuuk, Greenland

**Keywords:** syphilis, sexual transmitted diseases, venereal diseases, contact tracing

## Abstract

**Objective:**

To estimate the incidence of syphilis from 2010 to 2014 and to assess whether contact tracing has been performed.

**Study design:**

Observational cross-sectional study.

**Method:**

Data on reported cases were collected from the national register of the chief medical officer in Greenland. Unreported cases were found by searching the electronic medical record system for patients who had received an electronic prescription of benzathine penicillin, doxycycline or tetracycline. Medical records were reviewed to verify the diagnosis of syphilis and to evaluate if contact tracing had been performed.

**Results:**

Ninety-four cases of syphilis (51 males and 43 females) with a median age of 27 years (20–40) were included. The incidence of syphilis in Greenland has increased from zero cases in 2010 to 95.7 per 100,000 inhabitants in 2014 affecting mainly young people. Contact tracing was performed in 80.9% (76/94) of the cases.

**Conclusion:**

Syphilis has re-occurred in Greenland and a new epidemic may be underway. Sustained awareness of treatment, contact tracing, monitoring and preventive initiatives are desirable.

For decades, sexually transmitted diseases, such as gonorrhoea and chlamydia, have been highly incident in Greenland (1–6). Syphilis has been present in Greenland since at least the 1970s, with the first known epidemic reaching 746 cases in 1976. A second epidemic was seen in 1987 with 658 known cases (2), followed by a decline in incidence. For a number of years, approximately one case was reported each year (3).

In the past few years, local outbreaks have been observed, and in 2012 syphilis was made individually reportable to the chief medical officer (CMO) in Greenland. However, the actual incidence of syphilis remains unknown as well as contact tracing habits among health care professionals.

The aim of this study was to estimate the incidence of syphilis from 2010 to 2014 and to assess whether or not contact tracing has been performed.

## Methods

This study was conducted as an observational cross-sectional study based on register information and review of the electronic medical record system in Greenland. Data on reported cases from 2010 to 2014 were collected from the national register of the CMO in Greenland, and unreported cases were found by searching the electronic medical report system for patients who had received an electronic prescription of benzathine penicillin, doxycycline or tetracycline between 2010 and 2014. All medical records were reviewed to identify patients treated for syphilis. If the diagnosis of syphilis was recorded in the medical record of the case, the case was included as a case of syphilis, and if it was stated that contact tracing had been performed, it was included as such.

The incidence was estimated using the background population as of 1 January 2014.

The ethics committee for medical research in Greenland approved the study.

## Results

In total, 82 cases of syphilis were reported from the CMO. Three cases were excluded: one case was registered twice, one was only treated prophylactically as a primary contact, and one was registered incorrectly, as he or she had been treated for gonorrhoea, not syphilis. The search of the electronic medical record system resulted in 1,770 patients prescribed with benzathine penicillin, doxycycline or tetracycline. Of these, 66 had been diagnosed with syphilis out of which 51 cases were already reported from the CMO. This means that 15 cases had not been reported to the CMO by the health care professionals involved, indicating a rate of underreporting at 16% (15/94).

In total, 94 cases (51 males and 43 females) with a median age of 27 years (20–40) were included. No cases were diagnosed twice. The incidences are shown in Table I. The highest incidence was observed in 2014. Median age among males were higher than among females (p<0.001). The number of towns affected increased from one in 2011 to seven in both 2013 and 2014. In 80.9% (76/94) of the cases, contact tracing had been done and 33.0% (31/94) of the cases were diagnosed by contact tracing.

**Table I:** Gender-specific annual incidence of syphilis in Greenland per 100,000 inhabitants, including 95% confidence intervals and absolute numbers

Gender	Males	Females	p**	Total
Median age years (quartiles)	33 (22–44)	22 (19–30)	p<0.001	27 (20–40)
Year				
2010	No*	No*	–	No*
2011 (95% CI) (n/N)	6.7 (0–15.7) (2/29,730)	3.7 (0–11.0) (1/26,552)	0.631	5.3 (0–11.2) (3/56,282)
2012 (95% CI) (n/N)	19.5 (3.4–30.3) (5/29,730)	22.6 (6.7–38.5) (6/26,552)	0.624	19.5 (9.2–29.9) (11/56,282)
2013 (95% CI) (n/N)	50.4 (32.5–68.8) (15/29,730)	64.0 (45.8–82.3) (17/26,552)	0.500	56.85 (43.9–69.8) (32/56,282)
2014 (95% CI)s (n/N)	97.5 (92.0–103.1) (29/29,730)	71.6 (54.4–88.7) (19/26,552)	0.292	85.3 (76.0–94.5) (48/56,282)

* No cases observed

** Chi-test or Mann–Whitney test.

## Discussion

The incidence of syphilis in Greenland has increased from zero cases in 2010 to 85.3 per 100,000 inhabitants in 2014, despite contact tracing being performed in 80.9% of the cases. In comparison, the incidence of syphilis in Denmark was 6.25 per 100,000 inhabitants in 2013 (7). The reported incidence most likely represents a minimum national incidence since it cannot be excluded that some cases have neither been reported to the CMO nor have received an electronic prescription. Obviously, also subclinical and undiagnosed infections were not included. The incidence is highest among young people, who also have the highest incidence of gonorrhoea and chlamydia, which indicates high-risk sexual behaviour. Therefore, it is most urgent to stop the spread of syphilis. Increased awareness on symptoms, testing, treatment and contact tracing among health care professionals is needed. A national strategy based on an updated and re-implemented syphilis guideline, close centralized monitoring of new cases, contract tracing and after-treatment blood tests and feedback to clinicians is to be considered.

Involving the family and other resource persons in the management of the disease is desirable as a supplement to individual antibiotic treatment. A free and unprejudiced public debate on sexual habits involving teenagers and young people may help in prevention.
